# Age-Related Macular Degeneration: Clinical Findings following Treatment with Antiangiogenic Drugs

**DOI:** 10.1155/2014/346360

**Published:** 2014-02-16

**Authors:** Ricardo Casaroli-Marano, Roberto Gallego-Pinazo, Clemencia Torrón Fernández-Blanco, Marta S. Figueroa, Begoña Pina Marín, Gustavo Fernández-Baca Vaca, Antonio Piñero-Bustamante, Juan Donate López, José García-Arumí, Jordi Farrés Martí

**Affiliations:** ^1^Instituto Clinic de Oftalmología, Hospital Clínic de Barcelona, Universitat de Barcelona, Carrer Sabino Arana 1, 08028 Barcelona, Spain; ^2^Hospital Universitario y Politécnico La Fe, Bulevar Sur S/N, 46026 Valencia, Spain; ^3^RETICS Oftared, Instituto de Salud Carlos III, 28029 Madrid, Spain; ^4^Hospital Universitario Miguel Servet, Calle Isabel la Católica 1-3, 50009 Zaragoza, Spain; ^5^Hospital Universitario Ramón y Cajal, Carretera de Colmenar Viejo, Km. 9,100, 28034 Madrid, Spain; ^6^Hospital Dos de Maig de Barcelona, Carrer Dos de Maig 301, 08025 Barcelona, Spain; ^7^Hospital Regional Universitario Carlos Haya, Avenida Carlos Haya S/N, 29010 Málaga, Spain; ^8^Hospital Universitario de Valme, Avenida de Bellavista S/N, 41014 Sevilla, Spain; ^9^Hospital Clínico Universitario de San Carlos, Calle Profesor Martín Lagos S/N, 28040 Madrid, Spain; ^10^Hospital Universitario Vall d'Hebrón, Passeig Vall d'Hebron, 119-129 Barcelona, Spain; ^11^Bayer HealthCare, Avenida Baix Llobregat 3-5, Sant Joan Despí, 08970 Barcelona, Spain

## Abstract

*Purpose*. To survey the management of patients with neovascular age-related macular degeneration (nvAMD) in Spain. *Methods*. An observational retrospective multicenter study was conducted. The variables analyzed were sociodemographic characteristics, foveal and macular thickness, visual acuity (VA), type of treatment, number of injections, and the initial administration of a loading dose of an antiangiogenic drug. *Results*. 208 patients were followed up during 23.4 months in average. During the first and second years, patients received a mean of 4.5 ± 1.8 and 1.6 ± 2.1 injections of antiangiogenic drugs, and 5.4 ± 2.8 and 3.6 ± 2.2 follow-up visits were performed, respectively. The highest improvement in VA was observed at 3 months of follow-up, followed by a decrease in the response that stabilized above baseline values until the end of the study. Patients who received an initial loading dose presented greater VA gains than those without. *Conclusions*. Our results suggest the need for a more standardized approach in the management and diagnosis of nvAMD receiving VEGF inhibitors. To achieve the visual outcomes reported in pivotal trials, an early diagnosis, proactive approach (more treating than follow-up visits), and a close monitoring might be the key to successfully manage nvAMD.

## 1. Introduction 

Age-related macular degeneration (AMD) is the main cause of legal blindness (visual acuity lower than 20/200) in the Western world in people aged over 55 years [1]. It is a degenerative and progressive macular disease that results in loss of central vision with significant functional impairment. The most severe vision loss occurs in the neovascular form of AMD (nvAMD), involving choroidal neovascularization associated with retinal edema [2]. Even when AMD does not lead to blindness, there is a strong negative impact on independence and quality of life [3]. Worldwide, 25 million to 30 million people have severe visual loss due to AMD. The prevalence of AMD in Spain is estimated between 1.3% (65 to 74 years) and 5.7% (aged ≥75 years) of population, accounting for approximately 485.000 potential patients [4]. Since nowadays life expectancy is growing the prevalence of AMD is likely to increase [5], hence its diagnosis and treatment might represent an important challenge.

Therapies currently approved for nvAMD include laser photocoagulation, photodynamic therapy, and the new drugs capable of inhibiting vascular growth. The vascular endothelial growth factor (VEGF) inhibitors demonstrated improved visual outcomes compared with other therapies, becoming the first-line therapy for nvAMD [6]. The Phase III MARINA and ANCHOR trials with ranibizumab were designed with fixed monthly injections over a period of two years [7, 8]. However, as this treatment strategy is difficult to undertake in the regular clinical practice, other alternative approaches have been proposed by some retina specialist societies from several European countries, like the Spanish Society of Retina and Vitreous (SERV), recommending an initial loading dose followed by maintenance injections on an as-needed basis (PRN: *per re nata*) according to visual acuity and optical coherence tomography (OCT) changes [9]. Likewise, three German ophthalmologic societies [10] (der Retinologischen Gesellschaft, der Deutschen Ophthalmologischen Gesellschaft und des Berufsverbands der Augenärzte Deutschlands e.V.) and the prescription guidelines in France [11] recommend an initial 3-month upload phase of monthly injections and retreatment to prevent disease progression if there is evidence of disease activity or vision deterioration. Monitoring visits are recommended at monthly intervals following the upload phase in Germany [10] and in Spain [9]. Although some recent clinical practice studies have assessed the compliance with these guidelines [11–15], nothing has been published regarding the Spanish situation.

In order to face the future needs of nvAMD patients, it is necessary to broaden our knowledge about the correct management of the disease. Therefore, the aim of the present study was to survey the diagnosis and management of patients with nvAMD treated with antiangiogenic intravitreal therapy in terms of clinical practice in Spanish public health centers.

## 2. Methods

### 2.1. Patients

An observational retrospective study was conducted to assess the diagnostic management, therapeutic approach, and use of resources in Spanish public health centers for patients with nvAMD treated with ranibizumab or bevacizumab as first line. The inclusion criteria considered were (1) patients older than 18 years; (2) patients diagnosed with nvAMD on the first or second eye between February and June 2009; and (3) patients followed up since the diagnosis of nvAMD in the same center. Twelve ophthalmologists specialized in nvAMD from different tertiary care public Spanish hospitals participated in this study. Each one included the first 25 diagnosed cases that met the inclusion criteria. Patients were selected consecutively to avoid selection bias and each one was observed for a maximum period of two years. The selected eye for the analysis was the first eye diagnosed of nvAMD within this period. Whenever both eyes were diagnosed at the same visit, the studied eye was the one with better corrected visual acuity (VA). If VA was the same, the right eye was then selected.

The study was approved by the Spanish Agency of Medicines and Medical Devices (Agencia Española de Medicamentos y Productos Sanitarios) and the Ethical Review Board of each participating center.

### 2.2. Data

Patient characteristics were recorded at baseline. Clinical variables concerning the study eye were collected at baseline and at every follow-up visit, which could be treatment-related or not treatment-related. Baseline visits recorded the number of times that a diagnostic technique (biomicroscopy, fluorescein angiography (FA), and OCT) was used. The aim of each follow-up visit was to perform a clinical assessment of the patient: symptoms, lesion size (number of disc areas: <1, 1-2, >2), foveal thickness (measured in microns), macular thickness (measured in microns), and VA (measured with Snellen or ETDRS charts). The type of treatment and dose administered for nvAMD were also recorded, as well as the number of annual injections. The administration of an initial loading dose was considered as the injection of 3 doses in a period of maximum 60 ± 15 days between the first and third doses of ranibizumab or bevacizumab.

### 2.3. Statistical Analysis

To assess the evolution of clinical variables several temporal frames were defined: 3, 6, 12, and 24 months after the diagnosis of the study eye. The nearest follow-up visits to each temporal frame (±1 month) were included in the analysis of that frame.

Results were expressed as mean and standard deviation (SD) for the continuous variables and as the number and percentage of patients per category for categorical variables. The evolution of clinical variables was assessed by *t*-student or Cochran's-*Q* tests, depending on the variables characteristics (continuous or categorical, resp.). Early Treatment Diabetic Retinopathy Study (ETDRS) scores were converted to Snellen scores using a transformation table by Patel et al. [16]. Thereafter, Snellen fractions were approximately converted to letter count according to an equivalence table [17]. Data analysis was made using SPSS (Version 19) and SAS software (Version 9.2).

## 3. Results

### 3.1. Patient Baseline Characteristics

A total of 221 patients fulfilled the inclusion criteria and 208 were finally included in the analysis. The mean follow-up period was of 23.4 ± 4.2 months. A summary of the sociodemographic characteristics of the study population and the study eye is provided in Table 1. Of the 208 patients studied, 133 (63.9%) had nvAMD in only one eye and 75 (36.1%) in both. Smoking history was observed in 19.7% of patients: current smoker (5.8%) and former smoker (13.9%). The referral of patients was to general ophthalmologists or other retina specialists in 68.3% of cases, emergency services in 23.6% of cases and general practitioners or optometrists for the rest of the patients.

All patients conducted at least one of the diagnostic techniques assessed. A total of 95.7%, 76.4%, and 93.8% of patients underwent biomicroscopy, FA and OCT, respectively. Of note, 67.8% of cases underwent all the three techniques to establish the diagnosis of nvAMD.

Table 2 provides a description of the lesion and symptoms at baseline. The mean time between the appearance of symptoms and the diagnosis of the study eye was 1.9 ± 2.3 months. The administration of first-line treatment took place 14.9 ± 29.6 days after diagnosis in average.

### 3.2. Clinical Outcomes

Both foveal and macular thickness decreased over the study period. This change was already significant after 3 months of follow-up. The decrease of foveal thickness was in average 87.80 ± 85.88 *μ*m, 85.29 ± 97.74 *μ*m, 82.24 ± 104.01 *μ*m, and 83.73 ± 126.52 *μ*m at 3, 6, 12, and 24 months after diagnosis. The decrease of macular thickness was in average 34.81 ± 48.72 *μ*m, 37.16 ± 66.50 *μ*m, 35.26 ± 61.73 *μ*m, and 27.92 ± 56.44 *μ*m at 3, 6, 12, and 24 months after diagnosis. Thus, at baseline, the mean of foveal and macular thickness was 368.55 ± 125.14 *μ*m and 304.59 ± 63.41 *μ*m, respectively. And at the end of the study, mean foveal and macular thicknesses were 267.12 ± 104.18 *μ*m and 265.71 ± 30.70 *μ*m, respectively.

**Figure 1:**
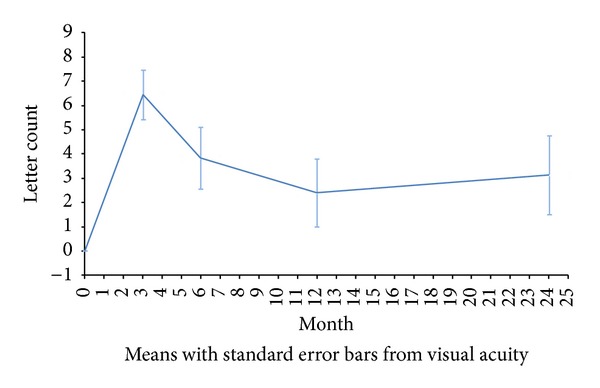
Changes in visual acuity over the follow-up period compared with visual acuity at baseline.

Mean VA gains of +6.45 ± 12.93, +3.84 ± 15.17, +2.41 ± 16.59, and +3.13 ± 19.64 letters were observed at 3, 6, 12, and 24 months of follow-up compared with baseline, respectively (Figure 1). The maximum improvement in VA was observed 3 months after diagnosis. Thereafter, the response decreased until one year of follow-up, when stabilized above the baseline values until the end of the study. Mean VA observed at baseline was of 47.93 ± 18.57 letters (0.25 or 20/80 approximately), and at the end of the study it was of 52.99 ± 22.24 letters (0.35 or 20/63 approximately).

Patients who were treated early after the diagnosis (less than 7 days) gained VA (+6.37 letters in average), whereas patients treated later than 14 days after the diagnosis lost VA (−4.41 letters in average) (*P* < 0.001).

### 3.3. Treatment

A total of 2802 visits were recorded, of which 1230 were treatment-related. At the end of the study, patients had received on average 6.1 intravitreal injections of antiangiogenic drugs: 4.5 ± 1.8 during the first year and 1.6 ± 2.1 during the second year. Overall, 14.7% of the visits were performed following the recommendation of monthly visits established by the SERV. During the first and second years of the study a mean of 66.0 ± 57.6 and 82.5 ± 53.1 days between consecutive follow-up visits was observed, respectively. A mean of 5.4 ± 2.8 and 3.6 ± 2.2 follow-up visits were performed per patient during the first and second year, respectively.

Most patients received ranibizumab (96.2%, *n* = 200) as antiangiogenic treatment, although an off-label use of bevacizumab was also reported (5.3%, *n* = 11). During the second year of follow-up about half of the patients (51.4%) received antiangiogenic treatment (50%, *n* = 104 with ranibizumab; 3.8%, *n* = 8 with bevacizumab) for nvAMD.

**Figure 2:**
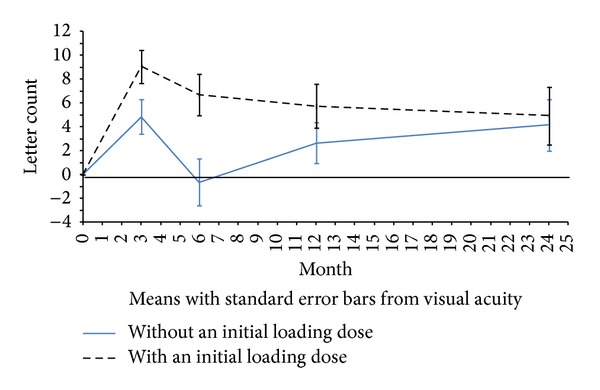
Changes in visual acuity depending on the administration of an initial loading dose.

A total of 105 patients (50.5%) did not receive a loading dose. Those patients with a loading dose (49.5%, *n* = 103) showed better results regarding final VA and decrease in foveal and macular thickness over the follow-up period. In particular, mean VA gains at 3, 6, 12, and 24 months of follow-up among the group of patients who received a loading dose were +9.06 ± 12.92, +6.69 ± 15.65, +5.75 ± 17.86, and +4.93 ± 20.31 letters, respectively; whereas the group without an initial loading dose gained in average +4.58 ± 13.30, −0.65 ± 15.54, +2.64 ± 16.22, and +4.14 ± 17.84 letters at 3, 6, 12, and 24 months of follow-up, respectively. Thus, at the end of the study both groups showed similar VA improvements (Figure 2). The difference reported at 6 months of follow-up between both groups was statistically significant (*P* = 0.0038).

Total injections received per patient were similar in both groups: a mean of 4.3 ± 2.1 and 1.6 ± 2.1 injections were recorded in the group without an initial loading dose during the first and second years, respectively, and a mean of 4.7 ± 1.8 and 1.6 ± 2.1 injections were recorded in the other group during the first and second years, respectively.

One particular participating center stood out among the others due to a more proactive treatment regimen, consisting of more treatment-related visits instead of follow-up visits. Over the study period this center performed a mean of 10.8 treatment-related visits per patient against 5.3 for the rest of the centers. Patients recruited (*n* = 33) in this hospital obtained substantially greater VA gains compared with patients of the rest of the centers.

## 4. Discussion

This observational retrospective study, performed in several public health centers in Spain, identified that the management of patients with nvAMD treated with VEGF inhibitors in routine clinical practice was variable and far away from that recommended by the European specialist guidelines in terms of treatment-related and not treatment-related visits. Likewise, the Lumiere study reflected poor compliance with treatment recommendations in France [11]. For example, the SERV Guideline and three German ophthalmologic societies recommend a loading treatment initiation followed by monthly follow-up visits to detect early recurrences and perform prompt retreatments to avoid as much as possible the permanent anatomic and functional damage [9, 10].

The results previously described showed that, at the end of the first year, patients gained in average only 2.4 letters from baseline and slightly more than 3 letters at the end of the study period. These gains are lower than those observed in the pivotal trials with fixed monthly regimen or strict PRN schemes with monthly visits [18, 19]. These differences can be explained by the wide variability found, which was related to time between first symptoms and diagnosis, time between diagnosis and initiation of treatment, and large follow-up visits intervals. The delay in the nvAMD diagnosis is likely to be caused by a lack of knowledge about the disease, but the treatment delay could be due to the different regional health systems and the accessibility to them. The large follow-up visit intervals and the fall of the use of antiangiogenic treatment observed during the second year of the study might be due to the big clinical burden in the ophthalmology departments of the public health system in Spain that makes a proper monitoring of the increasing nvAMD population more complicated.

The initial VA gain observed at the first 3 months was mainly attributable to patients who received an initial loading dose. Nevertheless, these patients gradually lost this greater gain and ended the follow-up period close to the gain achieved by the group of patients who did not receive the loading dose. This can be explained with the fact that, at the end of the study, both groups received a similar number of injections. As reported by the CATT research group [19], the effect of initial monthly doses disappears once the treatment regimen is not strict and proactive. The maximum improvement observed in VA at 3 months of follow-up is in accordance with other recent clinical practice studies in Sweden [12], Germany [13], Denmark [14], and France [11], where a peak of VA gain occurred after 3 months of treatment followed by a decrease of the response.

Few treatment administrations have been recorded in this study, and this fact could be explained by the low number of follow-up visits performed. As it has been reported in the CATT study [19], a PRN treatment approach for nvAMD patients could offer similar results to fixed monthly injections during the first year of treatment. Unfortunately, a monthly fixed regimen or monthly follow-up visits are hardly feasible in the current clinical practice due to the economic costs and/or the clinical burden in ophthalmology departments of the public health system in Spain. Therefore, further studies assessing the benefits and the costs associated to a stricter treatment regimen would be of interest.

It is worthy to mention the case of the public hospital with the highest rate of treatment-related visits. This center performs a more proactive treatment approach than the rest of the centers as more treating than monitoring visits are performed. In addition, VA outcomes obtained in this center are substantially better than the overall gains obtained in this study. Although one center out of 12 is not representative, the results found in this hospital support the findings reported in other studies, such us the MARINA [7] and ANCHOR [8] clinical trials that demonstrated efficacy of ranibizumab with monthly injections, and some recent studies, which demonstrated a higher response as the number of injections increases [20, 21]. In addition, this approach consisting in lower number of follow-up visits but more treatment visits could be affordable to obtain good VA results in overloaded health systems where monthly monitoring is not possible and the patient is exposed to large intervals between retreatment with VEGF inhibitor drugs, thus increasing the chance of disease reactivation and irreversible damage accumulation. Postponing retreatment until there is evidence of ongoing active disease might be leaving it too late [22].

Design limitations are those typically seen in retrospective studies, such as the lack of data in some medical records or the fact that the information is not directly collected from the patient. However, a retrospective design was the most appropriate in order to achieve the objectives of the present study. Another consideration is concerning the two scoring systems (Snellen and ETDRS charts), which are known to show differences, particularly in patients with nvAMD and poor VA [23]. Nevertheless, as values are shown in terms of changes in the scores, there is no risk of bias in the results.

In conclusion, our results show the need for a more standardized approach in the management and diagnosis of nvAMD patients treated with VEGF inhibitors in Spain. Other clinical practice studies [12, 13, 15] achieved the same conclusion in other European countries. An early diagnosis, a proactive approach, and close follow-up monitoring might be the key to successfully manage this degenerative disease. Earlier treatment including an initial loading dose followed by a stricter reinjection schedule could longer maintain VA gains in better values. An effort should be made to increase the adherence to the retina specialist guidelines in order to offer the best VA outcomes to nvAMD patients, while new treatments or nvAMD management are explored and validated. Avoiding reactivation of the disease should be considered as an aim of the nvAMD treatment instead of retreating disease reactivation, since a permanent damage is usually accumulated and never recovered, especially if the monitoring surveillance is not as often as it should be. Due to treatment cost and the significant impact that vision loss can have on quality of life, economic evaluations comparing current clinical practice and stricter reinjection regimens could bring valuable information in order to help the decision making process and to standardize a more appropriate treatment schedules.

## Figures and Tables

**Table 1:** Sociodemographic description of patients.

Variable	Total
Age (years)	
*n*	208
Average (SD)	76.72 (7.83)
Median	77
Age groups	
Total	**208 (100%)**
<65 years	14 (6.7%)
65–75 years	72 (34.6%)
>75 years	122 (58.7%)
Gender	
Total	**208 (100%)**
Male	88 (42.3%)
Female	120 (57.7%)
Ethnic group	
Total	**208 (100%)**
Caucasian	183 (88.0%)
Hispanic	25 (12.0%)
Iris color	
Total	**208 (100%)**
Light colored	44 (21.2%)
Dark	78 (37.5%)
Unknown	86 (41.3%)
Study eye	
Total	**208 (100%)**
1—left	105 (50.5%)
Secondary eye	
Total	**75 (100%)**
1—diagnosed before the study period	48 (64.0%)
2—diagnosed after the first study eye	15 (20.0%)
3—both eyes diagnosed at same time	12 (16.0%)

**Table 2:** Description of the lesion and symptoms at diagnosis.

Variable	Study eye—*n* (%)
Lesion type	
Total	**208 (100%)**
Classic	83 (39.9%)
Minimally classic	23 (11.1%)
Occult	71 (34.1%)
Other shapes	12 (5.8%)
RAP	9 (4.3%)
IPCV	3 (1.4%)
Unknown	19 (9.1%)
Lesion size	
Total	**208 (100%)**
<1 disk	38 (18.3%)
1-2 disks	90 (43.3%)
>2 disks	42 (20.2%)
Unknown	38 (18.3%)
Subretinal neovascular membrane location	
Total	**208 (100%)**
Yuxtapapillar	3 (1.4%)
Extrafoveal	22 (10.6%)
Yuxtafoveal	78 (37.5%)
Subfoveal	104 (50.0%)
Subfoveal + Yuxtapapillar	1 (0.5%)
Presence of symptoms at diagnosis	
Total	**208 (100%)**
Yes	208 (100%)
Symptoms at diagnosis	
Sudden and progressive loss of VA	136 (65.4%)
Central scotoma	88 (42.3%)
Difficulty to read	49 (23.6%)
Metamorphopsia	99 (47.6%)
Photopsia	1 (0.5%)
Other symptoms	2 (1.0%)
Other symptoms	
Total	**2 (100%)**

RAP: retinal angiomatous proliferation; IPCV: idiopathic polypoidal choroidal vasculopathy; VA: visual acuity.
